# A Compact Symmetric Microstrip Filter Based on a Rectangular Meandered-Line Stepped Impedance Resonator with a Triple-Band Bandstop Response

**DOI:** 10.1155/2013/457693

**Published:** 2013-11-11

**Authors:** Rajendra Dhakal, Nam-Young Kim

**Affiliations:** RFIC Lab, Department of Electronics Engineering, Kwangwoon University, Nowon-gu, Seoul 139-701, Republic of Korea

## Abstract

This paper presents a symmetric-type microstrip triple-band bandstop filter incorporating a tri-section meandered-line stepped impedance resonator (SIR). The length of each section of the meandered line is 0.16, 0.15, and 0.83 times the guided wavelength (*λ*_*g*_), so that the filter features three stop bands at 2.59 GHz, 6.88 GHz, and 10.67 GHz, respectively. Two symmetric SIRs are employed with a microstrip transmission line to obtain wide bandwidths of 1.12, 1.34, and 0.89 GHz at the corresponding stop bands. Furthermore, an equivalent circuit model of the proposed filter is developed, and the model matches the electromagnetic simulations well. The return losses of the fabricated filter are measured to be −29.90 dB, −28.29 dB, and −26.66 dB while the insertion losses are 0.40 dB, 0.90 dB, and 1.10 dB at the respective stop bands. A drastic reduction in the size of the filter was achieved by using a simplified architecture based on a meandered-line SIR.

## 1. Introduction

The triple-band bandstop filter (TBBSF) has evolved rapidly and has led to a dramatic demand for a lower-cost product with a compact size and strong communication capabilities. In a microwave communication system, the bandstop filter (BSF) is an important component that is typically adopted in the transmitter and receiver system. Triple-band antennas, baluns, and filters are required to accommodate triple-band wireless systems [1–3]. Triple-band BSFs are the key components for suppressing the specific bands of frequencies among these devices. Compared to single-band and dual-band filters, these filters are more popular due to their miniature size.

Recently, an increasing number of researchers have paid attention to triple-band bandstop filters as a key component for evolving WiMAX applications of the future. The conventional topology of filters design fails to meet the requirement of compact size [2–5]. Thus, this paper focuses on a design that expands the frequency band to reduce the size with a rectangular meandered-line stepped impedance technology. We proposed a symmetric-type rectangular meandered-line stepped impedance resonator (SIR) with triple-band bandstop characteristics. The tri-section stepped impedance concept is used to implement the desired BSF [5]. The proposed TBBSF has a total size reduction of approximately 82% and 67% compared with the conventional designs [2] and [3], respectively. This filter can be used to suppress the central resonant frequency (*f*_0_) by more than 25 dB in the stop bands and simultaneously achieve a wide stop band with a fractional bandwidth of 46.28%, 16.22%, and 8.05% for the first, second and third frequency bands, respectively. A comparative analysis between the symmetric and asymmetric TBBSF with respect to fractional bandwidth, loaded quality factor, and external quality factor is presented and verified with the necessary relevant figures and analysis.

## 2. Design and Theoretical Analysis

**Figure 1:**
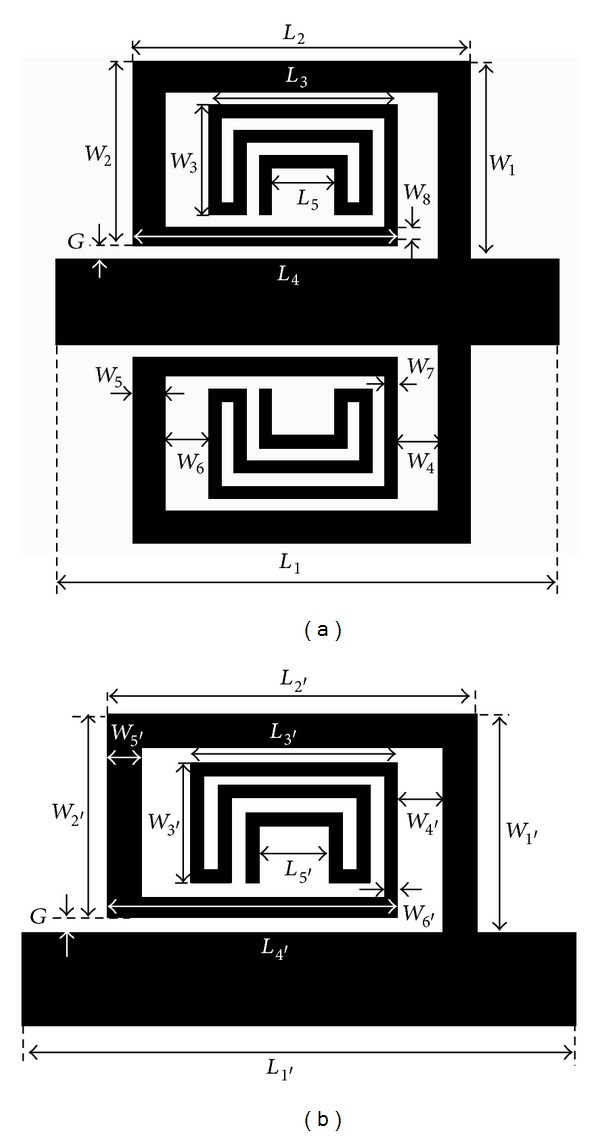
Design layouts of the two proposed TBBSFs: (a) symmetric, (b) asymmetric.

The proposed triple-band BSF is designed using a tri-section meandered-line open-end SIR with the length of each section being equal to 0.16, 0.15, and 0.83 times the guided wavelength (*λ*_*g*_) [6]. The schematic of the proposed symmetric triple-band BSF is shown in Figure 1. The meandered-line SIR is placed on the upper half of the planar transmission line to form an asymmetric BSF. We analyze the asymmetric BSF to compare it with a system requiring a relatively low bandwidth, a simple structure, and a higher quality factor than the symmetric BSF. However, for final verification of the design, we fabricated a symmetric-type TBBSF. To analyze the change in the fractional bandwidth (FBW) and quality factor between the symmetric and asymmetric structure, we perform a simulation analysis of the asymmetric TBBSF. Both of the structures generate a triple-band response with good selectivity and the same resonant frequency. The rectangular meandered line with different width is used to achieve the step impedance and placed symmetrically on either side of a 50 Ω planar microstrip transmission line with a folding coupling gap of *G*. The proposed structure is designed on a Teflon substrate with a thickness of 0.504 mm and a dielectric constant of 2.52.

### 2.1. Generalized Triple-Band Tri-Section Stepped Impedance Resonator

**Figure 2:**
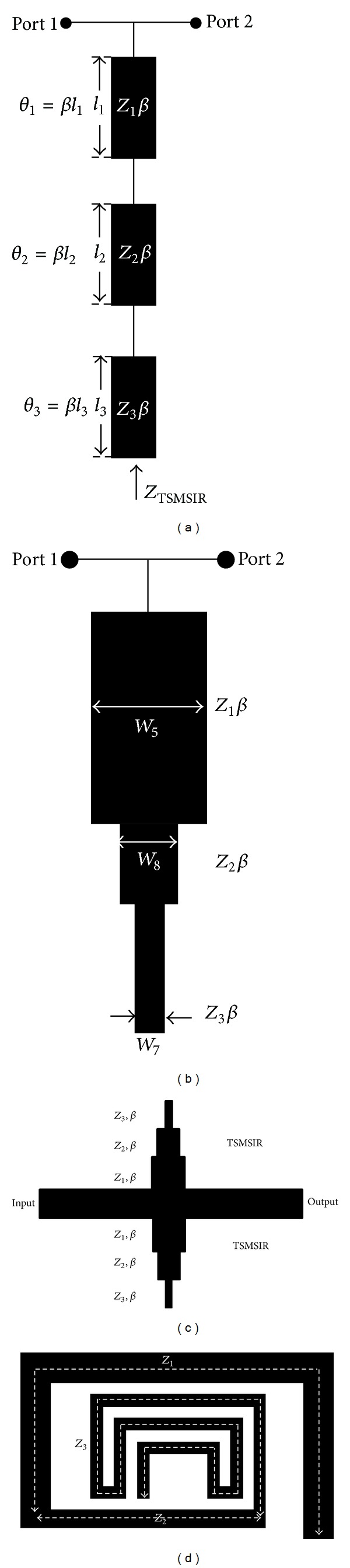
Configuration of (a) the general tri-section SIR, (b) the proposed SIR concept, (c) the proposed concept of a tri-section SIR, and (d) the proposed concept of a TSMSIR.

A TBBSF is formed by cascading 0.16*λ*_*g*_–0.15*λ*_*g*_–0.83*λ*_*g*_ SIRs to obtain a deeper skirt performance with triple-band bandstop characteristics. The SIR is comprised of low impedance *Z*_1_ section with electrical length of *θ*_1_ followed by high impedance *Z*_2_ section with electrical length of *θ*_2_ and a impedance *Z*_3_ section with electrical length of *θ*_3_.  Figure 2 shows the configuration of the tri-section meandered-line SIR (TSMSIR) with input characteristic impedance *Z*_TSMSIR_ [7–11]. The filter resonates at three different frequencies and can be used in the design of a TBBSF. Three different meandered-line structures with different characteristic impedances are implemented in a cascaded form to generate three distinct resonant frequencies and proved to be one of the best methods of reducing the size of the filter [12, 13]. The concept of SIR can be applied to implement multiband filters with the addition of other stepped impedance line. However, the insertion loss will degrade, and the topology will be too complex to bend the microstrip line. Additionally, the complexity of the junction discontinuity effects will be increased, so that we cannot obtain an accurate value of the insertion loss.

The layout of the proposed symmetric filter with the detailed dimensions is as follows: *L*_1_ = 10 mm, *L*_2_ = 6 mm, *L*_3_ = 3 mm, *L*_4_ = 4.55 mm, *L*_5_ = 1 mm, *W*_1_ = 3.2 mm, *W*_2_ = 3 mm, *W*_3_ = 1.74 mm, *W*_4_ = 0.95 mm, *W*_5_ = 0.5 mm, *W*_6_ = 1.05 mm, *W*_7_ = 2 mm, *W*_8_ = 0.36 mm, and *G* = 0.2 mm. The layout of the asymmetrical TBBSF is shown in Figure 1(b), and the detailed dimensions are as follows: *L*_1′_ = 10 mm, *L*_2′_ = 6 mm, *L*_3′_ = 3 mm, *L*_4′_ = 4.55 mm, *L*_5′_ = 1 mm, *W*_1′_ = 3.2 mm, *W*_2′_ = 3 mm, *W*_3′_ = 1.74 mm, *W*_4′_ = 0.95 mm, *W*_5′_ = 0.5 mm, *W*_6′_ = 2 mm, and *G* = 0.2 mm. The wavelengths corresponding to the three resonant frequencies *f*_1_ = 2.59 GHz, *f*_2_ = 6.88 GHz, and *f*_3_ = 10.67 GHz are *λ*_1_, *λ*_2_, and *λ*_3_, respectively. For simplicity, it is preferable to have equal electrical lengths for each section. So we set *θ*_1_ = *θ*_2_ = *θ*_3_ = *θ* = *π*/2 and *l*_1_ = *l*_2_ = *l*_3_ = *l*_0_ = *λ*_0_/4, where *λ*_0_ and *l*_0_ are the corresponding wavelength and length at the average frequency (*f*_0_) = 6.71 GHz, *β* is the propagation constant and, *θ* is the electrical length. The electrical length is given by

(1) $$\begin{array}{c} \theta_{1}=\beta_{1}l_{1}=\frac{2\pi }{\lambda_{1}}\times \frac{\lambda_{0}}{4}=\frac{\pi }{2}\times \frac{\lambda_{0}}{\lambda_{1}}\\ \theta_{2}=\beta_{2}l_{2}=\frac{2\pi }{\lambda_{2}}\times \frac{\lambda_{0}}{4}=\frac{\pi }{2}\times \frac{\lambda_{0}}{\lambda_{2}}\\ \theta_{3}=\beta_{3}l_{3}=\frac{2\pi }{\lambda_{3}}\times \frac{\lambda_{0}}{4}=\frac{\pi }{2}\times \frac{\lambda_{0}}{\lambda_{3}}. \end{array}$$

At resonance, the lowest impedance tri-section SIR exhibits a short termination. The admittance (*Y*) looking from the bottom portion of SIR shown in Figure 2(a) is given by

(2) Y=(Z3Z1−Z2Z1tanθ2tanθ3−Z2Z3tanθ1θ2−Z32tanθ1tanθ3)×(Δ)−1,

where

(3) Δ=jZ1Z2Z3tanθ2+jZ32Z1tanθ3+jZ3Z12tanθ1 −jZ2Z12tanθ1tanθ2tanθ3.

At resonance, *Y* = 0, which indicates that the final condition for resonance is

(4) $$\begin{array}{c} \frac{Z_{2}}{Z_{3}}\tan\theta_{2}\tan\theta_{3}+\frac{Z_{2}}{Z_{1}}\tan\theta_{1}\tan\theta_{2}+\frac{Z_{3}}{Z_{1}}\tan\theta_{1}\tan\theta_{3}=1. \end{array}$$

For impedance,

(5) $$\begin{array}{c} \frac{1}{Z_{i}}=Y_{i}=0. \end{array}$$

When *θ*_3_ = 0, the structure can be used as a two-impedance type SIR [14, 15]. If the electrical length is assumed to be equal, then the condition for the fundamental resonance of a tri-section SIR is given as

(6) $$\begin{array}{c} \theta =\text{ta}{\text{n}}^{-1}\sqrt{\frac{K_{1}K_{2}}{K_{1}+K_{2}+1}}, \end{array}$$

where *K*_1_ = *Z*_3_/*Z*_2_ and *K*_2_ = *Z*_2_/*Z*_1_. The resonator total length at the fundamental resonance is given by

(2.1) $$\begin{array}{c} \theta_{T}=3\theta =\text{ta}{\text{n}}^{-1}\sqrt{\frac{K_{1}K_{2}}{K_{1}+K_{2}+1}.} \end{array}$$

By selecting the appropriate values of *Z*_1_, *Z*_2_, and *Z*_3_, we can obtain the corresponding values of *θ*_1_, *θ*_2_, and *θ*_3_. Thus, both the length and the impedance ratio must be taken into account during SIR design.

### 2.2. Equivalent Circuit

**Figure 3:**
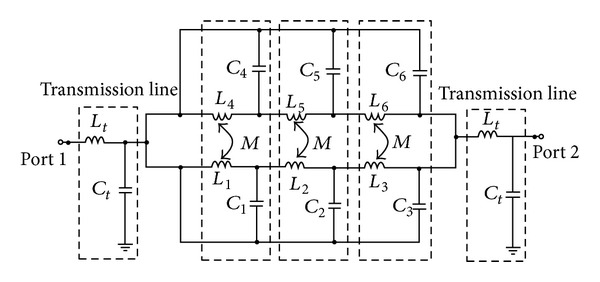
Equivalent circuit model of the symmetric TBBSF.

The equivalent circuit of the symmetric TBBSF is illustrated in Figure 3. Each SIR is equivalent to an LC tank circuit, and they have inductive coupling due to the meandered SIR above and below the transmission line. The LC tank circuits are cascaded together to generate three resonant frequencies. The filter resonates at three resonant frequencies *f*_1_, *f*_2_, and *f*_3_ represented by the three dashed blocks shown in Figure 3. When the series inductor (*L*_1_) and parallel capacitor (*C*_1_) resonate at *f*_1_, the input signal is shorted and opened, respectively, to form a stop band. When the series inductors *L*_2_ and *L*_3_ and parallel capacitors *C*_2_ and *C*_3_ have a very high and low impedance, they do not affect the input signal. A similar operation proceeds when the resonator *L*_2_, *C*_2_ and *L*_3_, *C*_3_ resonate at *f*_2_ and *f*_3_, respectively. Therefore the circuit operates as a TBBSF.

**Figure 4:**
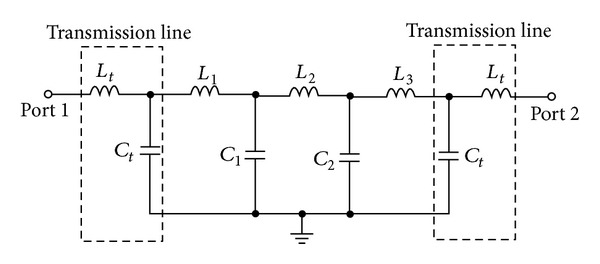
Equivalent circuit model of the asymmetric TBBSF.

Two similar structures on either side of the 50 Ω planar transmission line help to generate a wide bandstop bandwidth. Due to the magnetic coupling between the two structures, the total inductance (*L*_Total_) will be equal to the mutual inductance (*L*_*M*_) between them and the individual inductance of the meandered line in parallel. The resonant frequency can be determined using (8). Due to the increase in the inductance

(8) $$\begin{array}{c} f_{0}=\frac{1}{2\pi \sqrt{L_{\text{Total}}C}}, \end{array}$$

where *L*_total_ = (*L*_1_//*L*_4_) + *L*_*M*_ and *C* = *C*_1_//*C*_4_ value, the bandwidth that was determined using (9) eventually increases. The FBW is calculated by

(9) $$\begin{array}{c} \text{FBW}=\frac{f_{\max}-f_{\min}}{f_{0}}, \end{array}$$

where *f*_max⁡_ and *f*_min⁡_ are the band edge frequencies to the −3 dB return loss. The same operational principle is implemented in the asymmetric TBBSF shown in Figure 4 to obtain the equivalent circuit model. The asymmetric structure also possesses an LC tank circuit above the transmission line. The three LC tank circuits are cascaded together to generate the triple-band bandstop characteristics with a sharp roll-off. The total inductance in the circuit is due to the equivalence of the inductors in the LC tank circuit. The asymmetric structures possess less inductance than the symmetric filter. Due to the decrease in inductance, the bandwidths of the filter get decreased. Hence, the FBW and the external quality factor (*Q*_ext_) have been analyzed for the symmetric and asymmetric structures.

### 2.3. Quality Factor

In this paper, we propose a comparative approach of analyzing the loaded (*Q*_*L*_), unloaded (*Q*_*u*_), and external (*Q*_ext_) quality factors of the symmetric and asymmetric TBBSF and verifying these factors with the relevant relations and necessary figures. The *Q*_ext_ can be obtained from the loaded *Q*_*L*_ and the insertion loss of the filter at the resonant frequency. The *Q*_*L*_ value determines the sharpness of the transmission coefficient in the bandstop filter. The loaded and external quality factors are calculated and verified with an electromagnetic simulation. A higher value of *Q*_ext_ narrows the resonance response and lowers the feed line loss. For accurate measurements of the *Q*_*u*_, the coupling should be weak so that the loss of the feed line structure does not have a strong influence on the unloaded *Q*_*u*_ extrapolation. The asymmetric TBBSF has almost half of the bandwidth of the symmetric TBBSF with a sharp roll-off of the return loss response. The calculated external quality factor (*Q*_ext_) for the asymmetric structure is larger than that for the symmetric structure, which confirms the narrowness of the response and the lower signal loss in the circuit. The implementation of symmetric and asymmetric structures allows us to obtain an adaptable bandwidth phenomenon. Hence, a distinctly wide bandstop ability with a sharp roll-off is achieved with symmetric structure, whereas narrow bandstop characteristics can be obtained from the asymmetric structure separated by a transmission line. The unloaded quality factor (*Q*_*u*_) is calculated using the loaded quality factor and the insertion loss and is characterized by (10)

(10) $$\begin{array}{c} Q_{u}=\frac{Q_{L}}{1-S_{11}\left(f_{0}\right)}. \end{array}$$

The previous equation shows that, for a lossless system, *S*_11_ → *∞*, so that *Q*_*u*_ will be finite because of the inherent losses of the filter. The loaded quality factor (*Q*_*L*_) of the symmetrical structure is characterized by (11)

(11) $$\begin{array}{c} Q_{L}=\frac{f_{0}}{f_{\max}-f_{\min}}. \end{array}$$

The loaded quality factor for the asymmetric TBBSF is nearly twice that of the symmetrical TBBSF and is related by *Q*_*L*_′ = 2*Q*_*L*_. The required external quality factor (*Q*_ext_) of the filter can be calculated using (12)

(12) $$\begin{array}{c} Q_{\text{ext}}=\frac{f_{o}}{\text{FBW}}. \end{array}$$

**Figure 5:**
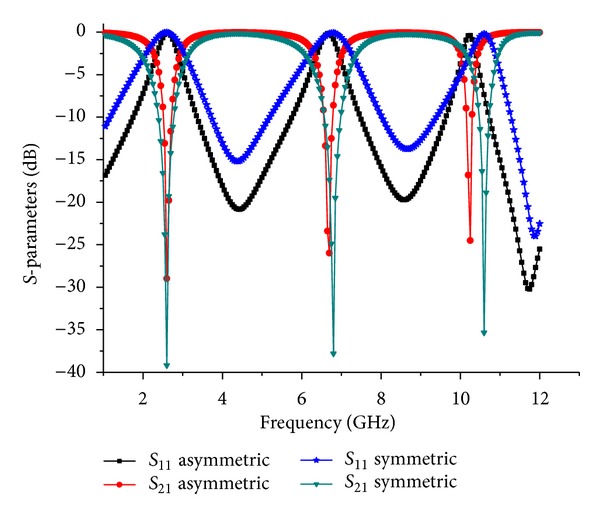
Simulated results of the symmetric and asymmetric TBBSFs.

**Figure 6:**
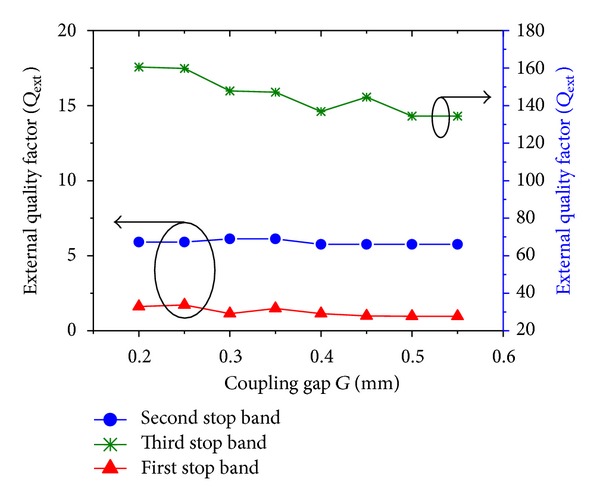
Behavior of *Q*_ext_ with the corresponding folding coupling gap *G* of the symmetric TBBSF.

The filters with symmetric and asymmetric TBBSFs were simulated with the help of the EM simulator SONNET. The *S*-parameters of those filters obtained from the simulations are displayed in Figure 5. The graph indicates the clear variation of the bandwidth between the two structures with nearly the same resonance frequencies. To assess the performance of the filter and the losses in the circuit, we performed an analysis of *Q*_ext_ and *Q*_*L*_. The dependency of *Q*_ext_ on the folding coupling gap (*G*) for the symmetric TBBSF was simulated, and the results are presented in Figure 6. The value of *Q*_ext_ for the first, second, and third bands slightly decreased as the folding coupling gap increased from 0.2 mm to 0.55 mm. This is because the increasing gap causes less coupling of the signal from the transmission line to the resonator. Therefore the coupling of the signal to and from the meandered line and the transmission line has been varied slightly. Additionally, we concluded that, as the gap increases, the signal loss for the third band is more prominent. Therefore, during the design of the multiband BSF using the stepped impedance meandered line, we must consider the decrease in the insertion loss [16–19].

**Figure 7:**
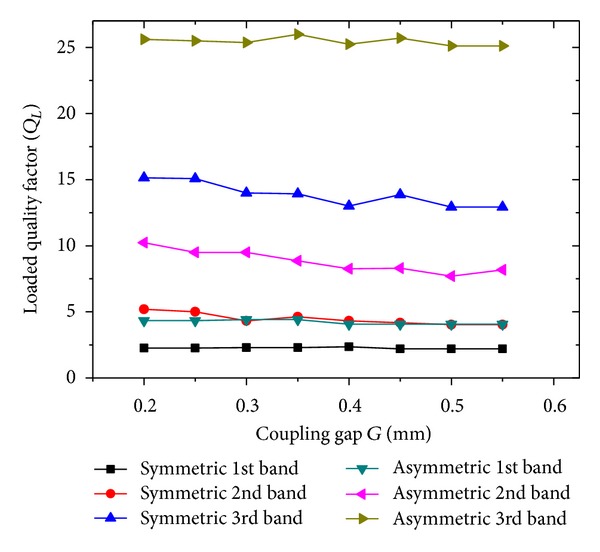
Variation of the loaded quality factor (*Q*_*L*_) of the symmetric and asymmetric TBBSFs with the corresponding folding coupling gap *G*.

Additionally, to make a comparison of the symmetric and asymmetric filters in terms of the FBW, we analyzed the loaded quality factor (*Q*_*L*_) as the folding coupling gap was changed from 0.2 to 0.55 mm. From Figure 7 we have verified that *Q*_*L*_ remains almost constant as the gap increases from 0.2 mm to 0.55 mm. The value of *Q*_*L*_ for the symmetric BSF is almost twice that for the asymmetric BSF. The *Q*_*L*_ hardly varies with the folding coupling gap, and, in the view of the high *Q*_ext_, the value of *G* was chosen to be 0.2 mm for further simulations.  Table 1 summarizes the comparison of the theoretical performance of the symmetric and the asymmetric TBBSFs.

From Table 1, it is clear that the external quality factor (*Q*_ext_) of the asymmetric structure exceeds that of the symmetric structure. Due to the implementation of the symmetric TBBSF, the bandwidth of the filter can be widened to almost double the value of the asymmetric TBBSF. Similar results can be obtained in the simulation for both the symmetric and asymmetric TBBSFs with the same first and second resonant frequencies at 2.59 GHz and 6.88 GHz, while for the third stop band the symmetric and asymmetric structures operate at 10.62 GHz and 10.27 GHz, respectively. Additionally, the stop band insertion loss for both of the filters is no more than 1.0 dB. The 3 dB bandwidths of the symmetrical BSF were 1.10, 1.17, and 0.72 GHz and were confirmed to be nearly twice that of the asymmetrical BSF for each band, thereby yielding higher *Q*_*L*_ than the symmetric BSF. The value of *Q*_ext_ indicates that the coupling of the signal from feed line is nearly constant and has little or no effect on the quality factor of the TBBSF with the variation of the folding coupling gap.

## 3. Implementation and Measurement

**Figure 8:**
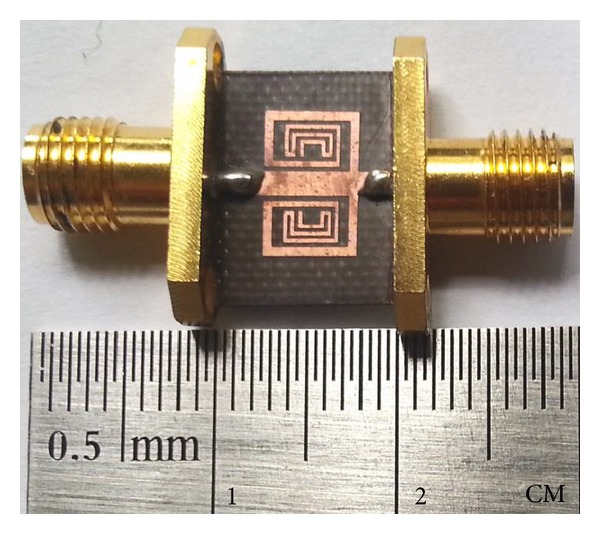
Photograph of the proposed TBBSF.

**Figure 9:**
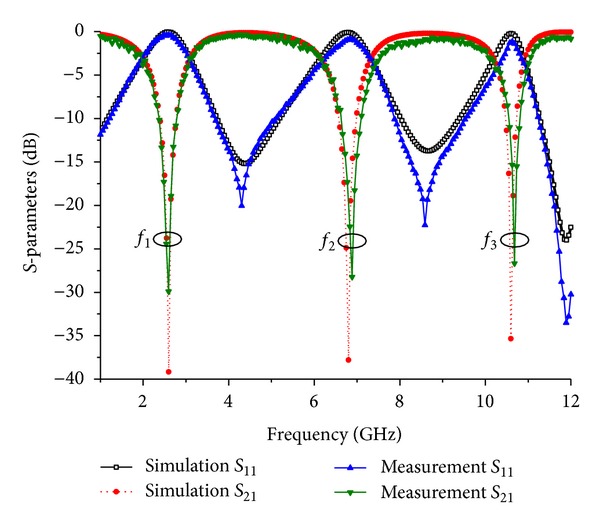
Measurement and simulation result of the proposed symmetric TBBSF.

To verify the proposed concept of a TBBSF, the symmetric-type BSF was fabricated using a Teflon substrate and then measured using an Agilent 8510C VNA. The fabricated filter has a total dimension of 10 mm by 6.40 mm as shown in Figure 8, and the measured *S*-parameter response of the filters is shown in Figure 9. The resonance frequencies of the filter were measured to be *f*_1_ = 2.59 GHz, *f*_2_ = 6.88 GHz, and *f*_3_ = 10.67 GHz with wide stop bands of bandwidths 1.12, 1.34, and 0.89 GHz for the first, second, and third bands, respectively. Thus, the results match the EM simulation well. The insertion loss of the stop band is 0.40, 0.90, and 1.10 dB for the respective resonant frequencies. The measurement result of the symmetric-type TBBSF shows a good agreement with the simulation results and is suited to be used as a BSF in WiMAX applications. The slight deviation observed in the measurements was attributed to the unexpected tolerances in fabrication and soldering the ports, which were not modeled during the simulation of the proposed filter.  Table 2 summarizes the simulation and measurement results of the proposed symmetric BSF. By comparing the design with the other literature shown in Table 3, we have obtained a significantly miniaturized size and a good insertion using a relatively lower value of the dielectric constant and substrate thickness.

## 4. Conclusions

 A triple-band bandstop filter incorporating a symmetric-type meandered-line step-impedance resonator and featuring wide bandstop characteristics has been designed and successfully fabricated with a significantly reduced size of 10 mm by 6.40 mm. A maximum size reduction of 82% in comparison to previous reports was achieved by bending the high impedance line along the width. The filter features three stop bands at 2.59 GHz, 6.88 GHz, and 10.67 GHz with high selectivity and good return loss response of −29.90 dB, −28.29 dB, and −26.66 dB at the respective stop bands. The measured results show a good agreement with the simulated results, exhibiting strong rejection of the signal in the three stop bands. The proposed filter has the advantages of a compact and miniature size, a sharp and deep skirt with good frequency selectivity in the vicinity of the resonance frequencies and is suitable for WiMAX applications. 

## Figures and Tables

**Table 1:** Performance of the symmetric and asymmetric TBBSF.

Parameter	Symmetrical simulation	Asymmetric simulation
Resonant frequency (GHz)	2.59, 6.80, 10.62	2.60, 6.70, 10.27
Loaded quality factor (*Q*_*L*_)	2.36, 5.91, 15.14	4.33, 10.54, 25.62
External quality factor (*Q*_ext_)	5.91, 32.89, 160.60	11.30, 70.52, 270.26
3 dB bandwidth (GHz)	1.10, 1.17, 0.72	0.60, 0.64, 0.4
Insertion loss (dB)	−0.18, −0.20, −0.30	−0.05, −0.20, −0.33

**Table 2:** Comparison of simulation and measurement results of the proposed symmetric TBBSF.

Parameter	Simulation	Measurement
Resonant frequency (GHz)	2.59, 6.80, 10.62	2.59, 6.88, 10.67
Insertion loss S_11_ (dB)	−0.08, −0.10, −0.16	−0.40, −0.90, −1.10
Return loss S_21_ (dB)	−39.16, −37.79, −35.33	−29.90, −28.29, −26.66
FBW at −3 dB (%)	42.47, 17.20, 6.77	46.28, 16.22, 8.05
Loaded quality factor (*Q*_*L*_)	2.36, 5.91, 15.14	2.31, 5.21, 11.48
External quality factor (*Q*_ext_)	5.91, 32.89, 160.60	6.02, 36.20, 122.64

**Table 3:** Comparison of performance characteristics with similar BSF and BPF studies.

Reference	Resonant frequency (GHz)	Insertion loss (dB)	Substrate *ϵ*_*r*_, *h* (mm)	Size (mm × mm)
This work	2.59, 6.88, 10.67	−0.40, −0.9, −1.10	2.52, 0.504	10 × 6.40
Literature [2]	2.37, 3.54, 5.01	−0.41, −0.35, −0.50	2.55, 1.5	36.3 × 10.04
Literature [3]	2.40, 3.50, 5.20	−1.5, −2.54, −1.16	2.2, 1.57	19.8 × 9.9
Literature [4]	2.43, 3.32, 5.60	−2.5, −1.8, −0.8	10.2, 1.27	30 × 10
Literature [5]	1.50, 2.57, 3.83	−2.2, −1.0, −1.5	3.38, 0.812	11.4 × 13.3
